# Dramatic Transformation After Burosumab in a Young Boy With X-linked Hypophosphatemia: A Life-Changing Saga

**DOI:** 10.7759/cureus.22340

**Published:** 2022-02-17

**Authors:** Krishna Baradhi

**Affiliations:** 1 Nephrology, University of Oklahoma School of Community Medicine, Tulsa, USA

**Keywords:** fibroblast growth factor-23, x-linked, burosumab, rickets, hypophosphatemia

## Abstract

X-linked hypophosphatemia (XLH), also referred to as vitamin D-resistant rickets or X-linked dominant hypophosphatemic rickets, is a very rare metabolic disorder. Despite its rarity, it is the most common form of genetic rickets. XLH is caused by loss of function mutation in the phosphate-regulating endopeptidase homolog X-linked (PHEX) gene, resulting in excessive fibroblast growth factor 23 (FGF23) activity. The end result is renal phosphate wasting leading to hypophosphatemia. It is frequently misdiagnosed as nutritional rickets as it mimics clinical manifestations of vitamin D-deficient rickets; however, it remains refractory to vitamin D repletion. The clinical expression can be variable from progressive bowing to severe skeletal and dental abnormalities. Treatment was limited to calcitriol and phosphate supplementation until the emergence of burosumab, a humanized monoclonal antibody against FGF23. We here share our first-hand experience of the use of burosumab in a 14-year-old boy with XLH and how it dramatically improved his quality of life along with the review of the literature regarding XLH and burosumab.

## Introduction

X-linked hypophosphatemia (XLH) is a complex hereditary bone disorder affecting early childhood and progressing into adulthood with multiple skeletal complications. XLH is dominantly inherited and arises due to mutations in the phosphate-regulating endopeptidase homolog X-linked (PHEX) gene that upregulates fibroblast growth factor 23 (FGF23). The FGF23 suppresses sodium phosphate co-transporter in the proximal tubule resulting in renal phosphate wasting and ultimately hypophosphatemia. Presenting symptoms usually include rickets to osteoarthritis, bone pain, enthesopathy, and dental complications. Traditional therapies with phosphorus repletion and calcitriol improved bone mineralization to some extent but never corrected the underlying pathophysiological mechanism until the arrival of the FGF23 inhibitor. Burosumab is a novel monoclonal antibody against FGF23. It mitigates renal phosphate wasting and improves serum phosphorus levels in patients with XLH. We describe the use of burosumab in a 14-year-old boy with XLH and review the current literature regarding this rare entity and treatment with this novel drug.

## Case presentation

A 14-year-old Caucasian male presented to our clinic to establish ongoing care for his rickets. His history was significant for childhood XLH. He originally presented as a child with leg pain and bowing of the legs with associated hypophosphatemia. He was referred to a bone and metabolic specialist and further testing confirmed urinary wasting of phosphorus. He was further evaluated and found to carry mutations in the PHEX gene, leading to the diagnosis of XLH. His family history was notable for his mother having XLH but with minimal symptoms and also his elder brother carried the same mutation.

He was started on oral phosphorus supplementation and calcitriol; however, his course was complicated with hyperparathyroidism and nephrocalcinosis. Calcitriol dose was augmented to suppress the parathyroid hormone (PTH) and citrate supplementation was also started due to hypocitraturia and nephrocalcinosis. Overall, he was taking close to 12 pills every day with a significant pill burden and impaired quality of life at the time of presentation to our clinic.

**Figure 1:**
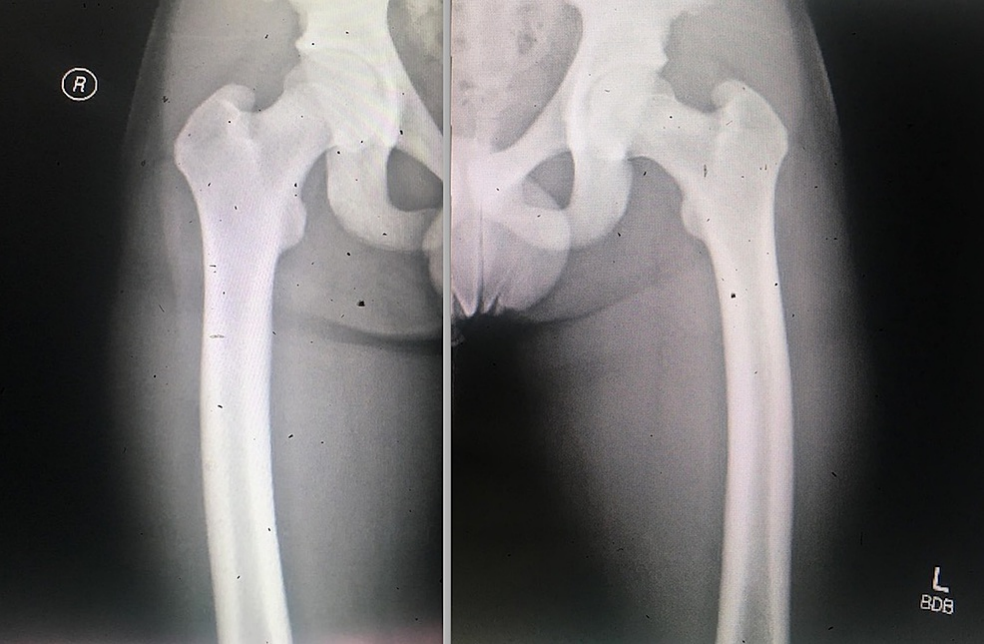
X-ray displaying symmetrical minor lateral bowing of each femoral shaft.

**Figure 2:**
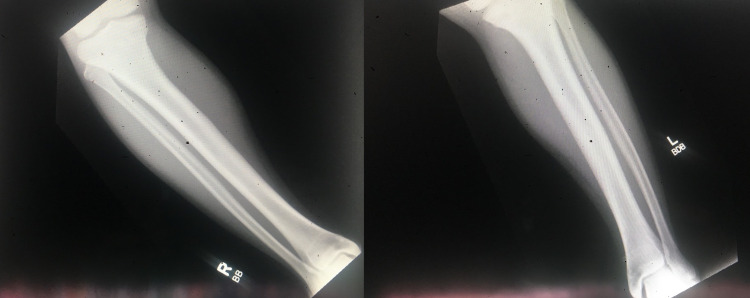
X-ray of the right and left tibia and fibula revealing tibial varus deformity.

Physical examination revealed a young boy with a normal cardiopulmonary examination; however, with an elevated blood pressure of 140/80 mmHg. Musculoskeletal examination divulged bowing of the legs predominantly seen on standing and while ambulating. Radiographs of bilateral lower extremities reveal the symmetric appearance of minor lateral bowing of each femoral shaft along with the tibia varus deformity without any other acute osseous changes (Figures 1, 2).

**Figure 3:**
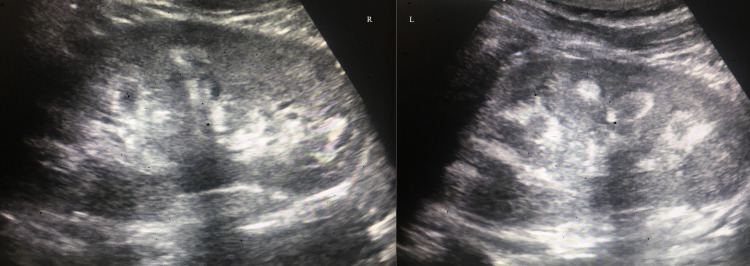
Renal ultrasound depicting increased density throughout the medullary pyramids compatible with medullary nephrocalcinosis.

Laboratory evaluation showed normal kidney function with a creatinine level of 0.67 mg/dL along with calcium of 9.1 mg/dL, albumin of 4.1 g/dL, and phosphorus of 2.2 mg/dL. The intact PTH level was 157 pg/ml and the vitamin D 25 level was 27 ng/mL. Renal ultrasound revealed a kidney size of 11.8 cm bilaterally with increased density throughout the medullary pyramids consistent with nephrocalcinosis (Figure 3). The patient was continued on calcitriol and phosphorus supplementation along with the addition of cholecalciferol to correct total vitamin D levels with close follow-up for elevated blood pressure. Subsequent office visits continued to show persistently elevated blood pressure despite dietary modification and the patient was eventually started on lisinopril to control his hypertension.

**Figure 4:**
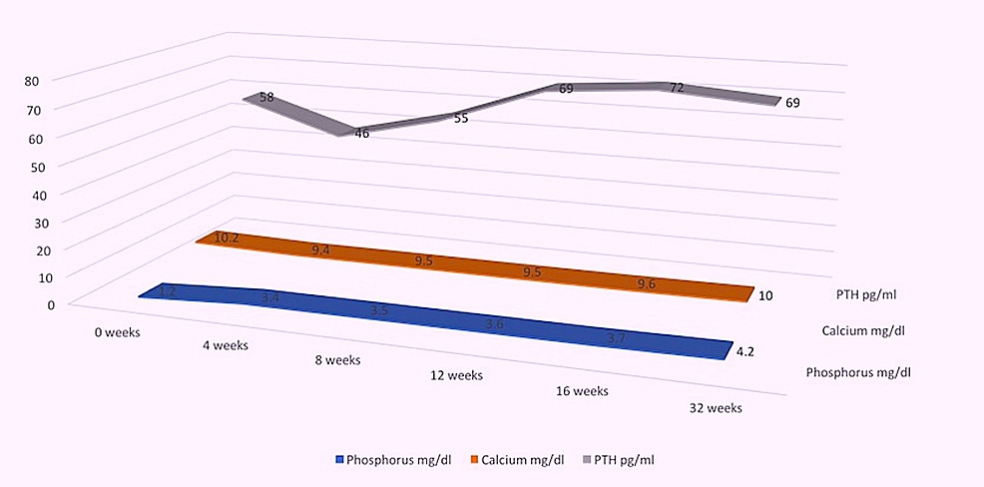
Calcium, phosphorus, and PTH levels before and after initiating burosumab. PTH, parathyroid hormone.

The patient's pill burden exceeded more than 15 pills per day, impairing his quality of life, until the advent of the new FGF23 inhibitor, burosumab. Burosumab was initiated after the washout period of one week, stopping all vitamin D and phosphorus supplements. Serum phosphorus level was 1.2 mg/dl prior to the commencement of the FGF23 inhibitor. Burosumab was initiated at 0.8 mg/kg subcutaneously every two weeks with close laboratory surveillance every two to four weeks. Burosumab was readily approved by the insurance after the required prior authorization was completed. Within four weeks after initiation of burosumab, his phosphorus levels normalized to 3.4 mg/dL and maintained normal phosphorus levels along with normal calcium levels (Figure 4). The patient's pill burden was drastically reduced to one pill/day, which significantly improved his quality of life. The only medication he was taking was lisinopril to control his blood pressure along with burosumab. His energy levels and intermittent musculoskeletal pains also improved.

## Discussion

XLH is a rare genetic disorder affecting one in 20,000 individuals with a female to male ratio of 2:1 [1]. XLH has an estimated prevalence of 3,000 children and 9,000 adults in the United States. XLH is the most familiar form of hereditary rickets [2]. Other hereditary rickets includes autosomal dominant and autosomal recessive hypophosphatemic rickets through mutations in FGF23 and dentin matrix protein 1 (DMP1), respectively. XLH is inherited in a dominant pattern with women having a 50% chance of transmitting this condition to their children, whereas men with XLH will pass this entity to all of their daughters, but not to their sons.

XLH occurs due to the incapacitating mutation of the PHEX gene located on the X-chromosome [3]. PHEX is a type I cell surface zinc metalloprotease and its mutations cause increased levels of circulating FGF23, though the exact mechanism is unclear. The FGF23 gene is located on chromosome 12p13 and is primarily expressed in bone osteocytes. FGF23 acts through fibroblast growth factor (FGF) receptors in conjunction with klotho protein [4]. FGF23 principally acts as a phosphaturic hormone by inhibiting sodium-dependent phosphate co-transporter 2a (NaPi2a), resulting in hypophosphatemia. FGF23 also hinders 1α-hydroxylase activity in the proximal tubule, resulting in low levels of active 1,25 vitamin D [5]. This sequel further causes hypocalcemia and PTH activation.

XLH primarily manifests as rickets, though fully penetrant, it has variable severity. The classical manifestation of bowing of the legs due to femoral and tibial bowing results in varus deformity, but at times can cause a valgus deformity as well, along with widening of the growth plates. These lower extremity abnormalities are predominantly noticed during ambulation with onset from the age of weight-bearing. These skeletal features may progress into gait deformities, slow growth velocity, and short stature. Generalized enthesopathy can occur in 69% of the affected individuals by the third decade [6]. Early-onset osteoarthritis is ubiquitous in affected adults along with osteomalacia. Chiari malformations due to flattening of cranial base occur in 44% of patients [7]. Periodontitis and dental abnormalities may occur due to dentin and cementum mineralization defects. Muscle weakness, bone pain, fatigue, and pseudo fractures may also complicate the clinical course [8]. Though life span is not necessarily affected by XLH, it is associated with significant morbidity and economic burden.

Hypertension is a characteristic complication of XLH [9]. The exact mechanism for developing hypertension is elusive though commonly associated with hyperparathyroidism [10].

Nephrocalcinosis is frequently seen in XLH and is often considered a consequence of phosphorus supplementation [11]. Hyperparathyroidism also occurs in XLH and is thought to be due to hypocalcemia and resultant stimulation of PTH, predominantly at night [12]. Chronic kidney disease may eventually ensue due to the above complications in some adults.

XLH often mimics nutritional rickets but it remains resistant to vitamin D therapy, hence called vitamin D-resistant rickets. Scurvy can rarely mimic rickets and needs to be considered in the differential diagnosis [13]. Other differential diagnosis includes autosomal dominant and autosomal recessive rickets due to DMP1 and ectonucleotide pyrophosphatase/phosphodiesterase 1 (ENPP1) mutations and X-linked recessive rickets from CLN5 mutations.

Diagnosis usually relies on the clinical presentation of rickets, hypophosphatemia, and urinary phosphate wasting. Alkaline phosphatase levels are typically elevated. PTH levels are usually in the upper limit of the normal range, while serum calcium levels are low to normal and urine calcium is low. Serum 1,25-dihydroxyvitamin D (1,25(OH)2D) levels are relatively low for the level of hypophosphatemia [14]. Renal ultrasound and skeletal imaging may further aid diagnosis. Definitive diagnosis is by genetic testing; however, evaluating for PHEX mutation is generally reserved for patients without a family history, atypical presentations, or for genetic counseling. FGF23 levels may be low, as they are influenced by several elements, and hence testing is not routinely advocated.

Conventional therapy focuses on correcting hypophosphatemia by phosphate supplementation. However, this can lead to transient hypocalcemia and resultant hyperparathyroidism, which exacerbate renal phosphate wasting. Combined calcitriol and phosphate therapy can offset some of these complications and improve bone mineralization. Usual recommendation is 20-40 mg/kg/day of elemental phosphorus and 20-30 ng/kg/day of calcitriol. Phosphate repletion can sometimes be limited by diarrhea. Standard therapies require meticulous lab monitoring and significant pill burden hampering the quality of life. Traditional treatments fail to correct underlying pathophysiology and are most often complicated with nephrocalcinosis and hyperparathyroidism. Despite these shortcomings, calcitriol and phosphate supplementation remained the standard of care, for the last four decades until the advent of novel FGF23 antagonists.

Burosumab, a fully humanized recombinant monoclonal Ig G1 antibody against FGF23 has changed the landscape in the treatment of XLH. Burosumab binds to FGF23 and inhibits its signaling, thereby allowing phosphate reabsorption and improving serum phosphorus and 1,25(OH)2D levels. FDA approved burosumab for the treatment of XLH for adults and children one year of age and older in April 2018. Burosumab is currently indicated for all pediatric patients with XLH and adults with ongoing symptoms, asymptomatic fractures, and complications.

Burosumab is typically initiated subcutaneously at 0.8 mg/kg/dose every two weeks in children and 1 mg/kg/dose every four weeks with dose titration as needed to achieve normal phosphorus levels. Following its absorption subcutaneously, burosumab has almost 100% bioavailability. It is degraded into small peptides and the meantime to reach maximum serum levels is 7.0 to 8.5 days with a mean half‐life of 16.4 days [15]. The apparent volume of distribution is 8L and elimination is not dose-dependent. All oral phosphorus and vitamin D analogs are discontinued for at least one week prior to initiation of burosumab. Serum phosphorus surveillance every four weeks while on therapy for the first 12 weeks and thereafter as appropriate is recommended per the manufacturer labeling. A positive correlation between serum burosumab and serum phosphorus levels advocates dosing based on predose phosphorus levels [15]. Burosumab is generally well tolerated with a limited side effect profile. Common adverse effects noted in the first 24 hours are vomiting, fever, rash, headache, dizziness, myalgia, and injection site reactions. Specific adverse events include hyperphosphatemia, hypersensitivity, and restless leg syndrome. Caution is advised in pregnancy, as burosumab may cross the placenta; however, no teratogenicity was reported in animal studies.

The efficacy of burosumab as the treatment for XLH in children was shown in an open-label trial conducted by Carpenter et al. [16]. A total of 52 children were randomly assigned in this study in a 1:1 ratio to receive burosumab every two or four weeks. The primary endpoint was the change in baseline Thacher rickets severity total score at 40 and 64 weeks. Studies showed therapy with burosumab not only improved serum phosphorus levels but also improved linear growth and physical function, and decreased severity of rickets. Whyte et al. conducted a similar study in 13 children aged one to four years with XLH and showed burosumab increased serum phosphorus levels, decreased severity of rickets, and prevented growth decline at 64 weeks [17].

Efficacy of burosumab treatment in symptomatic adult XLH patients was recently shown in a 24-week double-blinded, placebo-controlled, randomized trial conducted by Insogna et al. [18]. In this study, 134 symptomatic adults with XLH were randomly assigned in a 1:1 ratio to burosumab every four weeks or placebo. The study concluded that treatment with burosumab not only improved serum phosphorus levels but also positively impacted osteoarthritis indices and fracture healing when compared to placebo. Cheong et al. reproduced similar results in a multicenter, sequential dose-escalation, open-label, single-dose study in Japanese and Korean adults with XLH [19].

Our patient with XLH had poor quality of life while on calcitriol and phosphate therapy along with intermittent fatigue and bone pain. Moreover, he developed nephrocalcinosis, hyperparathyroidism, and hypertension. Initiation of burosumab was indeed life-changing for this teenager as it significantly enhanced his quality of life, improved fatigue, and bone pain, while drastically reducing his pill burden. Burosumab has definitely shown to be a promising drug and has revitalized the lives of children with XLH.

## Conclusions

XLH is a complex and rare genetic disorder that requires meticulous management and expert consultation. It is caused by elevated FGF23 levels due to PHEX mutations that result in urinary phosphate wasting and hypophosphatemic rickets. Conventional management with calcitriol and phosphate corrected serum phosphorus levels and bone mineralization but failed to correct underlying pathophysiology and anticipated complications. Burosumab offers a more optimal therapy when compared to conventional therapy with limited side effects, while significantly improving the quality of life.
